# Epigenetic memory and cell fate reprogramming in plants

**DOI:** 10.1002/reg2.73

**Published:** 2017-02-01

**Authors:** Kenneth D. Birnbaum, François Roudier

**Affiliations:** ^1^ Center for Genomics and Systems BiologyNew York University12 Waverly Place, New YorkNY10003USA; ^2^ Institut de Biologie de l'Ecole Normale Supérieure, Centre National de la Recherche Scientifique (CNRS) UMR8197Institut National de la Santé et de la Recherche Médicale (INSERM) U1024Ecole Normale Supérieure, 46 rue d'Ulm, 75230 Paris Cedex 05France; ^3^ Laboratoire de Reproduction et Développement des Plantes, Centre National de la Recherche Scientifique (CNRS) UMR5667, Institut National de la Recherche Agronomique (INRA) UMR879, Ecole Normale Supérieure de LyonUniversité Lyon 1 (UCBL)46 Allée d'Italie, 69364 Lyon Cedex 07France

**Keywords:** cellular reprogramming, chromatin remodeling, DNA methylation, epigenetic memory, plants, polycomb group complex, regeneration

## Abstract

Plants have a high intrinsic capacity to regenerate from adult tissues, with the ability to reprogram adult cell fates. In contrast, epigenetic mechanisms have the potential to stabilize cell identity and maintain tissue organization. The question is whether epigenetic memory creates a barrier to reprogramming that needs to be erased or circumvented in plant regeneration. Early evidence suggests that, while chromatin dynamics impact gene expression in the meristem, a lasting constraint on cell fate is not established until late stages of plant cell differentiation. It is not yet clear whether the plasticity of plant cells arises from the ability of cells to erase identity memory or to deploy cells that may exhibit cellular specialization but still lack an epigenetic restriction on cell fate alteration.

## 1 INTRODUCTION

Plant cells are well known for their endogenous plasticity as they can regenerate lost body parts from many different organs (Birnbaum & Alvarado, 2008). During regeneration, plant cells can transition from one specialized cell identity to another redefining their fate (Efroni et al., 2016; Sugimoto, Jiao, & Meyerowitz, 2010). Thus, plant cells must be good at either erasing or bypassing cell fate memory, or regeneration may simply employ cells that have little lasting memory of their fate (Sugimoto, Gordon, & Meyerowitz, 2011).

In addition to transcription factor networks that can stabilize cell fate through feedback regulation, mechanisms that act on chromatin are prime candidates for stabilizing cell fate, which, at least in some instances, could provide a memory system (Alabert et al., 2015; Barth & Imhof, 2010; Nashun, Hill, & Hajkova, 2015). Chromatin level modulation of gene expression relies on DNA methylation, covalent modifications of histones, incorporation of histone variants, as well as modifying and ATP‐dependent nucleosome remodeling enzymes (Feng & Jacobsen, 2011). Combinations of these epigenetic mechanisms translate into dynamic chromatin states with distinct transcriptional outcomes. Some chromatin modifications are only transient whereas others can be inherited through replication. Of particular interest here are mechanisms that could persist independently of the initial signal and therefore serve as a memory of cell fate.

Thus, a conceptual distinction should be made between chromatin level regulation that is immediately responsive to other inputs versus mechanisms that can perpetuate an epigenetic state without the need for a constant input (i.e., a cell memory, Barth & Imhof, 2010). For instance, lysine acetylation is associated with transcriptional activation and shows fast kinetics, whereas trimethylation at lysine 27 of histone H3 (H3K27me3) that is catalyzed by polycomb repressive complex 2 (PRC2) leads to a repressive state with a much lower turnover rate (Alabert et al., 2015; Barth & Imhof, 2010). Importantly, propagation of H3K27me3 across DNA replication is achieved by continuous modification of both parental and newly incorporated histones; such inheritance of chromatin state after cell division could serve as one form of cellular memory (Alabert et al., 2015). Finally, DNA methylation (5mC) is a key epigenetic mark which, once established, can be stably transmitted and is essential to silence transposable elements and mediate gene expression (Law & Jacobsen, 2010).

On a practical level, cell memory rarely appears to be completely independent of a cell's environment. Just as chromatin state can affect transcription, transcriptional regulation can modify chromatin state, as has been clear in mammalian cells where four transcription factors can reprogram cell fate and dramatically alter chromatin state (Nashun et al., 2015; Smith, Sindhu, & Meissner, 2016).

Indeed, the potential existence of an epigenetic memory function in plants implies that mechanisms would be needed to “unlock” or reset persistent chromatin states during cellular reprogramming. This could be achieved either actively by enzymes that remove chromatin modifications or passively through replication‐coupled dilution. For example, with respect to the latter, recent evidence in *Drosophila* indicates that, following replication, nucleosomes can displace transcription factors at expressed genes thereby resetting transcriptional states, whereas nucleosome positions are conserved at inactive chromatin (Ramachandran & Henikoff, 2016). Thus, DNA replication, during both mitotic and endoreplication cycles, could provide a strategic window of opportunity to modify the chromatin landscape, reset previous transcriptional programs, and rewire cell fate.

The question raised here is whether plant cells that participate in regeneration ever need their epigenetic memory to be erased, and, if so, at what stage of cell development does erasure become necessary? This phenomenon has a direct bearing on the concept of reprogramming and a mechanistic understanding of the phenomenon of “dedifferentiation.”

## 2 REGULATING THE EMBRYONIC TRANSITION THROUGH CHROMATIN

Chromatin dynamics have been implicated in the transition from youthful to differentiated states in both plants and animals. Mutations in the PRC2 components *CURLY LEAF* (*CLF*) and *SWINGER* (*SWN*) or in *FERTILIZATION INDEPENDENT ENDOSPERM* (*FIE*), the first two of which are homologous to the *Drosophila* protein Enhancer of zeste (E(z)) and the third to Extra sex combs (Esc), lead to somatic embryogenesis in the adult plant (Bouyer et al., 2011; Chanvivattana, Bishopp, Schubert, Stock, Moon, Sung, & Goodrich, 2004; Goodrich, Puangsomlee, Martin, Long, Meyerowitz, & Coupland, 1997). In addition, mutants impaired in components of PRC1, another type of polycomb repressive complex, lead to a similar phenotype (Merini & Calonje, 2015). Similarly, a mutation in *PICKLE* (*PKL*), which is a CHD3 chromatin‐remodeling factor that is also implicated in transcriptional repression, leads to a failure in the transition from embryonic to seedling state, showing ectopic expression of embryonic regulators, such as *LEAFY COTYLEDON2* (*LEC2*), in the adult phase (Ogas, Kaufmann, Henderson, & Somerville, 1999). Since ectopic expression of *LEC2* is sufficient to trigger the formation of somatic embryos from vegetative tissues (Stone et al., 2001), it is not clear whether the persistence of youth exhibited in compromised chromatin modification backgrounds is due to a global failure to shut down embryonic programs or to the ectopic transcriptional activation of one or a few pioneering factors like *LEC2* that can override the transition.

## 3 CHROMATIN LEVEL REGULATION IN LOCAL DEVELOPMENTAL TRANSITIONS

Recent work has also clearly demonstrated a role for chromatin in local developmental progressions in the root and shoot. In the shoot meristem, cells with high auxin levels initiate Aux/IAA proteolysis, which leads to the dislodgement of the co‐repressor TOPLESS‐HISTONE DEACETYLASE 19 (TPL‐HDA19) complex, thus enabling the recruitment of the SWI/SNF family members SPLAYED (SYD) and BRAHMA (BRM) (Wu, Yamaguchi, Xiao, Bargmann, Estelle, Sang, & Wagner, 2015). In turn, this remodeling of chromatin probably favors the binding of additional transcription factors and the recruitment of histone acetyltransferase activity to sustain the transcriptional activation of auxin responsive genes. Since regeneration usually entails the redistribution of auxin maxima, it is likely that such a chromatin switch could be key in early reprogramming events.

In the root, the protein of WUSCHEL‐RELATED HOMEOBOX 5 (WOX5) moves from the quiescent center to its distal neighbor to prevent its differentiation (Pi et al., 2015). WOX5 interacts with Groucho‐like TPL and related proteins as well as HDA19 to repress expression of genes that promote differentiation (Pi et al., 2015). In the opposite progression, ASYMMETRIC LEAVES forms a complex in the shoot that physically interacts with PRC2 to silence shoot meristem regulators in the differentiating leaves (Lodha, Marco, & Timmermans, 2013).

It is worth noting that the activity of chromatin modifiers responds to chemical environment and it is not clear if they persist independently of the signaling pathways in which they are implicated. For example, does the silencing of “youthful” transcriptional programs by epigenetic mechanisms ever need to be undone during regeneration?

## 4 CHROMATIN LANDSCAPE ALTERATION IN REGENERATION

In young tissues, like meristems, cells can respond to new positional signals that alter cell fate during regeneration in just a few hours (Efroni et al., 2016; Xu, Hofhuis, Heidstra, Sauer, Friml, & Scheres, 2006). However, in older tissues, regeneration is often a longer and less efficient process (Birnbaum & Sanchez Alvarado, 2008). For example, one common regeneration protocol requires incubation of mature tissue explants in high concentrations of the phytohormone auxin (callus inducing medium, CIM) for 4 to 10 days in order to form highly potent callus, a mass of tissue capable of generating roots and shoots (Skoog & Miller, 1957; Sugimoto & Meyerowitz, 2013). The lengthy competence step could reflect the time needed to alter chromatin and DNA modifications through either active mechanisms or a passive replication and dilution (Sun et al., 2014).

Studies investigating the role of chromatin changes in regeneration show involvement in both shutting down “old” cell fates and permitting upregulation of “new” cell fates during reprogramming from callus. One gene implicated in this transition, *METHYLTRANSFERASE 1* (*MET1*), mediates DNA methylation at CG sites (Kankel et al., 2003). *WUSCHEL* (*WUS*), which is necessary for stem cell function in the shoot meristem, is activated earlier during callus formation in *met1* compared to wild type. Consistently, bisulfite sequencing showed lower methylation at the *WUS* locus in the mutant (Li, Liu, Cheng, Su, Han, Zhang, & Zhang, 2011). In an emerging theme of epigenetic mechanisms having a role in hormone regulation, *AUXIN RESPONSE FACTOR 3* (*ARF3*) was also misregulated in the *met1* mutant. These effects in the *met1* background suggested a role for DNA methylation in regeneration but it was not clear if removal of DNA methylation at the *WUS* locus was necessary for regeneration.

However, another recent study implicated the removal of non‐CG methylation as a key step mediating regenerative competence during CIM incubation. The study utilized mutants in *DOMAINS REARRANGED METHYLTRANSFERASE 1* and *2* (*DRM1/DRM2*), and *CHROMOMETHYLASE 3* (*CMT3*) (Shemer, Landau, Candela, Zemach, & Eshed Williams, 2015), which, when combined, lack DNA methylation at non‐CG sites (CHG/CHH; Stroud et al., 2014). Explants from plants carrying mutated versions of all three genes (*ddc*) could skip the competence step, forming shoots in cytokinin rich shoot inducing medium (SIM) without pre‐incubation in CIM (Shemer et al., 2015). The study found that *WUS* could be directly activated by SIM in the *ddc* mutant but not in wild type. Also, the *ddc* mutant showed hypomethylation at the *WUS* locus, as with *met1*. The authors speculated that, in wild type, successive divisions on CIM and SIM led to the gradual dilution of DNA methylation as a result of downregulation of *CMT3* in the final stages (Shemer et al., 2015). Thus, the model proposes a passive mechanism to erase DNA methylation memory. An alternative is that the mutant changed the response to SIM independently of an effect on cellular memory. Still, the study provides one of the strongest pieces of evidence that induced callus formation requires the erasure of otherwise stable epigenetic information mediated by DNA methylation.

Several other studies have also shown dynamic regulation of DNA methylation in callus induction in trees (Vining et al., 2013) and during somatic embryogenesis in a variety of species (Nic‐Can, Lopez‐Torres, Barredo‐Pool, Wrobel, Loyola‐Vargas, Rojas‐Herrera, & De‐la‐Pena, 2013; Stroud et al., 2013; Teyssier et al., 2014; Viejo, Rodriguez, Valledor, Perez, Canal, & Hasbun, 2010). Thus, dynamic regulation of DNA methylation appears pervasive in regenerative processes in plants, although it is not clear in these studies whether methylation provides a lasting source of information for the cell.

In addition to DNA methylation, histone modification was also shown to have a role in the transition to callus from mature tissues. In auxin‐induced callus formation, CIM treatment instigates a transition through root identity before even shoots can be induced (Sugimoto et al., 2010). In a leaf to callus transition, mutations in two PRC2 components, *clf* and *swn*, led to defects in callus formation, while the root to callus transition was unaffected (He, Chen, Huang, & Xu, 2012). ChIP‐chip analysis in wild type showed that H3K27 was targeted to leaf specific genes; for example, *SAWTOOTH1* (*SAW1*) and (*SAW2*) were downregulated and also showed increased H3K27 methylation (He, Chen, Huang, & Xu, 2012). These results led the authors to speculate that the role of PRC2 was to help orchestrate the transition from leaf to root‐like callus by shutting down the leaf transcriptome (He et al., 2012).

Still, *SAW1* and *SAW2* were downregulated in the *clf swn* mutant 2 days after callus induction. The two genes did show a lack of repression compared to wild type later in the transition at 4 days after callus induction (He et al., 2012), suggesting that the role of PRC2 was to maintain repression rather than initiate it.

The maintenance role of PRC2 is consistent with an epigenetic memory function that needs to be removed to permit the induction of newly induced cell fates. Such a mechanism could target root genes that need to be induced during callus formation. A candidate erasure mechanism is the *RELATIVE OF EARLY FLOWERING 6* (*REF6*) gene, which was shown to recognize specific DNA sequences at H3K27me3‐marked genes (Cui et al., 2016; Li et al., 2016; Lu, Cui, Zhang, Jenuwein, & Cao, 2011). However, mutations in *REF6* did not lead to any measurable defects in leaf to callus formation (He et al., 2012). The result was inconclusive since redundancy in the *REF6* family may simply obscure a role in permitting the activation of new cell fate programs, or, alternatively, “unlocking” of H3K27 repression may not be required, at least in the leaf to callus transition.

## 5 A POTENTIAL LATE STAGE ROLE FOR EPIGENETIC CONTROL OF CELL IDENTITY

Recent studies suggest that PRC2 activity might provide a differentiation memory to prevent adult tissues from “slipping back” into earlier developmental programs once cells have left the signaling environment of the meristem. In the root, meristematic cells can undergo dramatic morphological changes in late differentiation, as shown by the tip growth and endoreplication of epidermal hair cells (Guimil & Dunand, 2007; Schiefelbein, 2000). In the *clf swn* double mutant, meristem patterning and identity showed no major defect during development, with root hairs developing normally. However, at a late stage, endoreplicated root hairs remarkably reinitiated cell division and reverted back to an embryo‐like development program (Ikeuchi et al., 2015). The defects in PRC2 repressive activity led to an upregulation of genes involved in wound response as well as embryonic regulators, such as *LEC2*. These data indicate that root hair differentiation does not depend on PRC2 activity, which, nonetheless, does seem to be required later to stabilize late stages of differentiation.

Interestingly, PRC2 was implicated in a somewhat similar “slipping back” phenotype in stomatal development. In wild type stomata, stem‐cell‐like meristemoid mother cells self‐renew and generate the precursors to the stomatal lineage (Bergmann & Sack, 2007). Within the lineage, guard mother cells differentiate into guard cells—a step mediated by the basic helix−loop−helix transcription factor *FAMA* (Ohashi‐Ito & Bergmann, 2006). It was shown recently that *FAMA*’s role in stabilizing the differentiated state appeared to work through *RETINOBLASTOMA‐RELATED* (*RBR*) and the PRC2 complex (Matos, Lau, Hachez, Cruz‐Ramirez, Scheres, & Bergmann, 2014). FAMA mutants that could not interact specifically with RBR reverted back from a guard mother cell precursor to the meristemoid stem cell state—a progression not seen in wild type cells (Matos et al., 2014). A separate study found that *FAMA* could mediate H3K27 methylation of stomatal stem cell genes (Lee, Lucas, Goodrich, & Sack, 2014). In addition, RB/RBR proteins are known to recruit chromatin modification complexes to the regions where they bind (Burkhart & Sage, 2008; Gutzat, Borghi, & Gruissem, 2012). It is not clear yet whether the FAMA/RBR interaction plays a role directly on chromatin to mediate H3K27 modification. Still, together, these results could suggest a model in which *FAMA* recruits a complex that includes PRC2 to lock down differentiated cell fate in stomatal development (Matos et al., 2014).

The two studies provide strong evidence that PRC2 can stabilize cellular differentiation independent of the early patterning mechanisms that establish cell fates since these phenotypes were observed after cells exited the meristem or progressed beyond early stomatal development. These studies did not specifically address the role of the PRC2 in regeneration, and it is not yet clear that the plant has or needs mechanisms to erase such an epigenetic control during endogenous regeneration. Root hairs and stomatal cells appear to represent quite stable differentiated fates, and these cells may never be employed for plant cell regeneration outside of the mutant phenotypes described above.

## 6 CONCLUSION

There is growing evidence in plants that epigenetic processes stabilize cell fate at least partially independently of early signals that specify fate. This appears to provide some memory of, at least, the developmental stage of a cell. Such a role is evident in recent studies showing that PRC2 stabilizes stomatal and root hair cell identity. For example, the switch from root hair to embryonic fate in the mutant PRC2 background occurred long after embryonic development was complete, presumably entailing ectopic activation of embryonic programs that are normally shut down. Indeed, chromatin level profiling shows that embryonic genes targeted by PRC2 are largely shut down in the adult root (Bouyer et al., 2011; Roudier et al., 2011). While alternative explanations are possible, it seems highly likely that, as long suspected, chromatin modification provides the highly plastic plant cell a way to stabilize tissue patterns generated in a signaling environment that the cell no longer perceives. This may take the form of a complete lockdown of cell fate or, perhaps more likely, each stage of development may be subject to temporary stabilization of gene expression programs that prevent “slipping back” to earlier developmental stages (Signolet & Hendrich, 2015). Memory need not be long term.

Still, regeneration studies have begun to question whether the broad array of cells shown to demonstrate totipotency using hormone treatments truly represents the type of cells that participate in regeneration under endogenous conditions. For example, in *Arabidopsis*, callus was shown to originate from one specific tissue, pericycle (Sugimoto et al., 2010). Cells within the root and shoot meristems and apparently the leaf cambium appear to be particularly competent to regenerate without hormone treatments (Feldman, 1976; Liu et al., 2014; Reinhardt, Frenz, Mandel, & Kuhlemeier, 2003). Thus, the plant has subsets of cells distributed throughout its body that may typically be employed during regeneration. It is not clear that such cells have a chromatin‐level memory that needs to be erased but this remains a pressing question in the field.

In pursuit of the role of epigenetic memory as a gatekeeper to cell fate, one fundamental step is determining the extent to which chromatin marks reflect cell identity on a global level. One recent study showed that cells in the root meristem did indeed exhibit cell type specific methylation patterns (Kawakatsu et al., 2016), but it is not yet clear if such epigenetic marks correlate with gene expression patterns. While cell culture lines in animals have provided a tractable approach to assay chromatin modifications in specialized cells, plants lack such an extensive collection of differentiated cell culture lines. The field will have to rely on primary cells (e.g., Deal & Henikoff, 2011)—perhaps a more accurate reflection of in vivo states but also a more challenging approach.

Overall, the evidence that persistent chromatin states present a barrier that the cell must overcome to regenerate is extremely limited, although the question is central to understanding the plant's high capacity to regenerate. Intriguingly, in mutants that lose almost all non‐CG methylation, a lengthy competence step typically required to induce regeneration through callus is no longer required, implying the competence step was necessary, in part, to alter the chromatin landscape. Such an erasure of methylation marks may occur through cell replication and dilution, as the study proposed, but it is not yet clear if reprogramming in plants requires cell division in any context. Most critically, to answer the question of whether dedifferentiation in plants requires an erasure of chromatin‐encoded memory, more evidence documenting the requirement to erase epigenetic marks during regeneration and cellular reprogramming is needed.
